# A Novel Metal Nanoparticles-Graphene Nanodisks-Quantum Dots Hybrid-System-Based Spaser

**DOI:** 10.3390/nano10030416

**Published:** 2020-02-27

**Authors:** Mariam M. Tohari, Andreas Lyras, Mohamad S. AlSalhi

**Affiliations:** 1 Department of Physics, College of Science, King Khalid University, P.O. Box 9004, Abha 62529, Saudi Arabia; 2 Department of Physics and Astronomy, College of Science, King Saud University, P. O. Box 11451, Riyadh 11451, Saudi Arabia; alyras@ksu.edu.sa (A.L.); malsalhi@ksu.edu.sa (M.S.A.); 3 Research Chair on Laser Diagnosis of Cancers, College of Science, King Saud University, Riyadh 11451, Saudi Arabia

**Keywords:** spaser, plasmonic amplifiers, graphene nanodisks, metal nanoparticles, quantum dots cascade emitters

## Abstract

Active nanoplasmonics have recently led to the emergence of many promising applications. One of them is the spaser (surface plasmons amplification by stimulated emission of radiation) that has been shown to generate coherent and intense fields of selected surface plasmon modes that are strongly localized in the nanoscale. We propose a novel nanospaser composed of a metal nanoparticles-graphene nanodisks hybrid plasmonic system as its resonator and a quantum dots cascade stack as its gain medium. We derive the plasmonic fields induced by pulsed excitation through the use of the effective medium theory. Based on the density matrix approach and by solving the Lindblad quantum master equation, we analyze the ultrafast dynamics of the spaser associated with coherent amplified plasmonic fields. The intensity of the plasmonic field is significantly affected by the width of the metallic contact and the time duration of the laser pulse used to launch the surface plasmons. The proposed nanospaser shows an extremely low spasing threshold and operates in the mid-infrared region that has received much attention due to its wide biomedical, chemical and telecommunication applications.

## 1. Introduction

Over the last decade, plasmonic nanosources have attracted significant attention as coherent and intense near-field generators of nanolocalized optical fields, eliminating the need for a coupling mechanism between photons and surface plasmons (SPs), and paving the way to many promising applications [1,2]. The surface plasmon waves, resulting from collective oscillations of free electrons near the surface of metals, propagate along the interface between metal and dielectric and decay in the direction perpendicular to the interface. The propagation length of surface plasmon polaritons is limited by the plasmonic losses that depends on the dielectric properties of metals [3]. For a metal bounded by an ideal dielectric, the losses are caused by free electron scattering and absorption through interband transitions at significantly shorter wavelengths [4].

Because the losses are often a serious limitation for practical applications of nanoplasmonics, many efforts have been devoted to proposing loss compensation and amplification approaches by introducing a gain medium in the dielectric surrounding the metal. Specifically, Seidel et al. reported the first experiment demonstrating amplification of surface plasmons on a flat silver film surrounded by dye molecules [5]. This work was followed by many theoretical and experimental investigations [6,7,8,9,10,11,12,13] that have paved the way to surface plasmon amplification by stimulated emission of radiation, i.e., spaser, introduced by Bergman and Stockman [14] and demonstrated experimentally with various plasmonic resonators, gain media and geometries [15,16,17,18,19]. Moreover, several theoretical approaches have been established to explain the spasing quantum mechanically, using two-level and three-level models for the gain medium [20,21]. Richter et al. have constructed a numerical approach to account for a large number of identical chromophores with an arbitrary number of energy levels non-perturbatively, based on density matrix theory through the use of the Tavis-Cummings model that has been introduced to describe the collective behavior of multiple atomic dipoles interacting with electromagnetic radiation [22].

Due to their ability to confine the optical energy near the surface, the spaser can utilize plasmonic components as its resonators to support the plasmonic modes and externally excited population-inverted gain media in order to provide the energy for spasing modes. The demonstration of spasing involves a resonant energy transfer from optical transitions in the gain medium to plasmon excitations in the metal, as well as a stimulated emission of surface plasmons due to the high local fields created by plasmons that excite the gain medium and stimulate more emission of selected plasmonic modes, leading to the required amplification [23].

The performance of a spaser is limited by the relaxation rates of the surface plasmons and the gain medium. To overcome the former limitation, plasmons in graphene provide a suitable alternative to those of metals due to the high mobility of its charge carriers, leading to tight confinement and a relatively long propagation distance, as well as the tunability of graphene’s plasmons via electrostatic gating [24,25,26,27]. A spaser formed by doped graphene nanoribbons surrounded by semiconductor quantum dots (QDs) has been theoretically proposed [11]. This spaser has been shown to support a wide frequency generation region from terahertz to infrared, small plasmon damping and a low pumping threshold. Moreover, a tunable spherical graphene spaser that supports localized surface plasmon modes has been proposed. It was found that the spasing could occur when the quality factors of some localized modes becomes larger than some critical values given in terms of the Fermi energy of graphene [13]. Additionally, Apalkov et al. have proposed a novel nanospaser made of a graphene nanopatch and a quantum well cascade emitter [12]. With this spaser, optical fields have been generated, exhibiting a high nanolocalization and coherent generation of SPs in the graphene nanopatch.

However, the quantum well cascade suffers from nonradiative relaxation of electrons in the upper radiative state, due to thermally activated electron-longitudinal optical phonon scattering [28]. Interestingly, due to the discrete nature of their energy levels, it is possible to greatly increase the lifetime of the upper levels of QDs via a phonon bottleneck that suppresses electron-longitudinal optical phonon scattering [29]. Thus, a spaser that utilizes a quantum dots cascade emitter as its gain medium could be characterized by a low spasing threshold [30]. Moreover, based on selection rules, the optical transitions in QDs cascade emitters are only allowed along the direction of the QDs growth [31]. This can significantly simplify the calculations of a spaser.

Recently, it has been shown that a metal nanoparticles-graphene nanodisks-quantum dots hybrid system can support the ultrafast energy transfer between excitons and plasmons [32]. Specifically, within the near field approximation, having a relatively large size of metal nanoparticles (MNPs) of polarizability comparable to that of highly doped graphene nanodisks (GNDs) that support plasmons that are resonant with excitons in the QD, leads to a controllable and ultrafast energy transfer within the system [32]. Therefore, it is expected that by using an MNP-GND hybrid system as a resonator, one can enhance the performance of a spaser and exercise more control on its performance characteristics, since the plasmons in graphene can be launched and controlled effectively with resonant metal nanoantennas [32,33,34].

Clearly, there is continuous interest in developing novel spaser devices with lower spasing thresholds, a simplified but robust structure, high amplification and stable performance. In the present work, we propose a novel nanospaser composed of an MNP-GND hybrid plasmonic system as its resonator and a quantum dots cascade as its gain medium to take advantage of the tunability of long-lived plasmons in graphene [27] and the possibility to control the graphene plasmons by plasmons in noble metals [33], as well as the low spasing threshold with a quantum dots cascade emitter. The proposed spaser operates in the mid-infrared region that has wide chemical, biomedical and telecommunication applications [35]. The properties of the spaser as a plasmonic amplifier will be investigated using the density matrix theory with a quantized plasmonic field in the transient regime.

## 2. Theoretical Formalism

**Figure 1:**
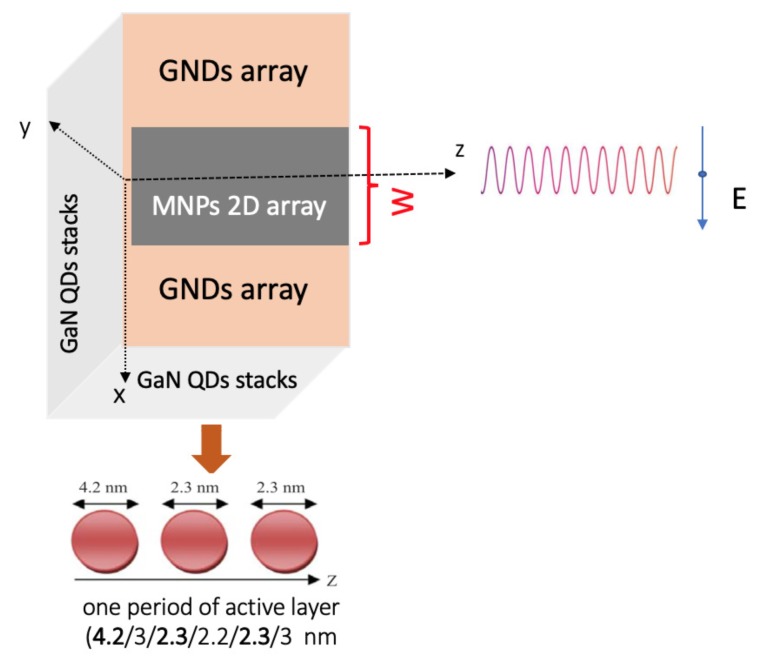
Schematic illustration of the proposed metal nanoparticle (MNP)-graphene nanodisk (GND)-quantum dots (QD)-based spaser.

We aim to study the properties of the proposed spaser depicted in Figure 1 that utilizes the MNP-GND hybrid system as its resonator and a gallium nitride (GaN) QDs cascade stack as its gain medium. The GaN QDs stack is characterized by mechanical and thermal stability, as well as low sensitivity to ionizing radiation [36]. Moreover, GaN QDs cascade terahertz emitters, modeled for one period shown in Figure 1, where the bold numbers denote the size of the QDs that are sandwiched between $Al_{0.18}Ga_{0.82}N$ layers, have demonstrated a relatively long lifetime of the upper level, leading to enhanced population inversion that supplies the spasing mode [37]. Let us first find the electric and magnetic field components of the plasmonic excitations near the GNDs arrays, taking into account the presence of the MNPs lattice. To this end, consider a two-dimensional periodic lattice of silver nanospheres of width *W*, on top of a periodic lattice of GNDs, located at z=0, encompassing the |x|<W/2 region and centered at x=0.

Consider a transform limited pulse propagating along the z-direction, with its linear polarization along the interface between the GNDs layer and the dielectric (i.e., along the *x*-axis). The launching pulse induces the fields $E^{ind}$ and $B^{ind}$ that obey the Maxwell’s equations, and due to the symmetry in the *y*-direction, the fields have transverse magnetic polarization (TM) with $E_{x}$, $E_{z}$ and $B_{y}$ nonzero field components. Thus, by using Fourier transformation, one can write the field induced along the *x*-direction as:

(1) $$E_{x}^{ind}\left(x,z,t\right)=\int_{-\infty }^{\infty }e^{iqx-i\omega t}E_{x}^{ind}\left(q,z\right)dq$$

where *q* is the wavenumber of the SPs waves propagating along the interface. In the following, the dependence on time will be dropped in order to simplify the notation and focus on the spatial dependence. Using Maxwell’s equations in the spectral representation, one can find:

(2) $$E_{x}^{ind}\left(q,z\right)=-i\frac{k_{q}}{2\epsilon_{0}\epsilon \omega \tilde{E}\left(\omega \right)}J_{x}\left(q\right)e^{-k_{q}\left|z\right|}$$

where $k_{q}^{2}=q^{2}-\epsilon \frac{\omega^{2}}{c^{2}}$, and ϵ is the dielectric constant of the embedding medium. Note that $\tilde{E}\left(\omega \right)$ represents the envelope of the pulse in its angular frequency domain. $J_{x}\left(q\right)$ is the Fourier component of the current density in the plane z=0, which can be written as:

(3) $$J_{x}\left(x\right)=\sigma_{GNDs}E_{x}\left(x,0\right)+\left(\sigma_{MNPs}-\sigma_{GNDs}\right)\Theta \left(\frac{W}{2}-\left|x\right|\right)E_{x}\left(x,0\right)$$

where Θ is the Heaviside step function and E(x,0) is the total electric field at z=0 given as the sum of the external and induced fields. $\sigma_{GNDs}$ and $\sigma_{MNPs}$ are the effective conductivity of GNDs and MNPs arrays, respectively. To model the optical response of nanoparticles arrays, it is fairly convenient to employ the effective medium approximation (EMA) that is only valid when the inner structure length scale is much smaller than the incident wavelength. Within EMA, the system comprising of interacting nanoparticles (NPs) is replaced by a homogeneous layer with an effective dielectric constant representing the overall response of the modeled system [38]. The effective dielectric constant for nanodisks and nanospheres arrays has been derived analytically by Genov et al. through considering the RLC model of NPs arrays, in which the negative permittivity of plasmonic material and the positive permittivity of the dielectric are represented by inductance R-L and capacitance C, respectively [39]. Based on this model for a plasmonic material, graphene (G) or metal (M), of a dielectric constant written as $\epsilon_{M,G}=\epsilon_{M,G}^{{}^{\prime }}\left(1-i\kappa_{M,G}\right)$, with $\kappa_{M,G}<<1$, where $\kappa_{M,G}$ represents the loss parameter that is given as the ratio between the relaxation rate of plasmons $\gamma_{M,G}$ and the incident frequency ω, the effective dielectric constants of the periodic arrays of GNDs and MNPs embedded in a dielectric of $\epsilon_{d}$ are given as:

(4a) $$\begin{array}{c} \epsilon_{GNDs}=\epsilon_{G}^{{}^{\prime }}\left[\frac{\pi }{2\kappa_{G}\left(\Delta_{G}-i\right)\left(P_{G}+1\right)}-\frac{1+\pi /2}{P_{G}+1}\right] \end{array}$$

(4b) $$\epsilon_{MNPs}=2\epsilon_{d}\left[\left(1+\frac{\kappa_{M}\Delta_{M}}{P_{M}+1}\right)log\left(\frac{P_{M}+1}{\kappa_{M}\left(\Delta_{M}-i\right)}\right)-1\right]$$

where $P_{M,G}=\left|\epsilon_{M,G}^{{}^{\prime }}\right|/\epsilon_{d}$, $\Delta_{M,G}=\left(\frac{P_{M,G}}{\delta_{M,G}}-1\right)/\kappa_{M,G}$. $\delta_{M,G}$ is the packing density of arrays given as a ratio between the diameter of particles and the interparticle distances, and with the approximation of plasma frequency ${\left(\omega_{p}\right)}_{M,G}>>\omega$, $\epsilon_{M,G}^{{}^{\prime }}$ as ${\left(\omega_{p}/\omega \right)}_{M,G}^{2}$.

Substituting from Equation (2) into Equation (1), through the use of the Fourier transformation of $J_{x}\left(x\right)$, leads to [40]:

(5) $$E_{x}^{ind}\left(x,z\right)=-\frac{i}{4\pi \epsilon_{0}\epsilon \omega \tilde{E}\left(\omega \right)}\int_{-\infty }^{\infty }dqk_{q}e^{iqx-ik_{q}\left|z\right|}\int_{-\infty }^{\infty }dx^{{}^{\prime }}J_{x}\left(x^{{}^{\prime }}\right)e^{-iqx^{{}^{\prime }}}$$

Combining Equations (5) and (3) leads to the following equation for $E_{x}\left(x,0\right)$ in all space [40]:

(6) $$E_{x}\left(x,0\right)=\frac{1}{2\pi \tilde{E}\left(\omega \right)}\left(1-\frac{\sigma_{GNDs}}{\sigma_{MNPs}}\right)\int_{-\infty }^{\infty }dq\frac{e^{iqx}}{\xi_{q}}\int_{-W/2}^{W/2}dx^{{}^{\prime }}e^{iqx^{{}^{\prime }}}E_{x}\left(x^{{}^{\prime }},0\right)+\frac{\sigma_{GNDs}}{\sigma_{MNPs}}\frac{E_{0}}{\tilde{E}\left(\omega \right)\xi_{0}}$$

where $\xi_{q}=1+\left(i\sigma_{GNDs}k_{q}/2\omega \epsilon_{0}\epsilon \right)$ is the dielectric function of the GNDs layer. Clearly, $E_{x}\left(x^{{}^{\prime }},0\right)$ involved in Equation (6), represents the field inside the metallic contact of width *W*, which can be written as a Fourier expansion:

(7) $$E_{x}\left(x^{{}^{\prime }},0\right)=\sum_{n=0}^{\infty }A_{n}cos\left(\frac{2\pi nx^{{}^{\prime }}}{W}\right)$$

Thus, Equation (6) can be reduced to a system of algebraic equations to be solved numerically in a matrix form running the sum up to a judicially chosen upper value n=N. After determining the coefficients $A_{n}$, the electric field induced along the x-direction near the graphene layer due to the inhomogeneity introduced by the metallic contact is obtained in terms of the dimensionless quantities u=qW and $k_{u}=k_{q}W$ as [41]:

(8) $$E_{x,u}\left(x,z\right)=\frac{2\zeta_{\sigma }}{\pi \tilde{E}\left(\omega \right)}\sum_{n=0}^{N}{\left(-1\right)}^{n}A_{n}\int_{0}^{\infty }du\frac{1-\xi_{u}}{\xi_{u}}\frac{usin(u/2)}{u^{2}-4n^{2}\pi^{2}}cos\left(ux/W\right)e^{-k_{u}\left|z\right|/W}$$

where $\zeta_{\sigma }=\left(\sigma_{MNPs}/\sigma_{GNDs}\right)-1$, and

(9) $$\xi_{u}=1+\frac{i\sigma_{GNDs}}{2\omega \epsilon_{0}\epsilon W}\sqrt{u^{2}-a^{2}}\;\mathrm{and}\;a^{2}=\frac{\epsilon \omega^{2}W^{2}}{c^{2}}.$$

It is clear that the parameter *a* contains a dependence on the ratio of the width of the metallic contact to the incident wavelength. Thus, *a* can be considered as a measure of raterdated effects. Specifically, the case of small *a*, i.e., u>a, corresponds to an evanescent wave confined near the graphene layer. Equation (8) gives the electric field component of the induced field along the interface. The transverse component can be found by using the following Maxwell’s equations valid for TM polarization:

(10a) $$\begin{array}{c} E_{z}^{ind}\left(x,z\right)=i\frac{c^{2}}{\omega \epsilon }\partial_{x}B_{y}^{ind}\left(x,z\right) \end{array}$$

(10b) $$B_{y}^{ind}\left(q,z\right)=i\frac{\omega \epsilon }{c^{2}k_{q}^{2}}\partial_{z}E_{x}^{ind}\left(q,z\right)$$

Using Equation (2), and substituting from Equation (10b) into Equation (10a) while inserting $J_{x}$ given through Equation (3), we obtain the two-dimensional electric field induced along the *z*-direction due to the metallic contact:

(11) $$\begin{array}{ll} E_{z,u}\left(x,z\right)= & -sgn\left(z\right)\frac{2\zeta_{\sigma }}{\tilde{E}\left(\omega \right)\pi }\sum_{n=0}^{N}{\left(-1\right)}^{n}A_{n}\int_{0}^{\infty }du\frac{1-\xi_{u}}{\xi_{u}}\frac{u^{2}sin\left(u/2\right)}{u^{2}-4n^{2}\pi^{2}}\frac{sin(ux/W)}{k_{u}}\\ & \times e^{-k_{u}\left|z\right|/W} \end{array}$$

The corresponding transverse component of the magnetic field can be obtained using Equation (10a):

(12) $$\begin{array}{ll} B_{y,u}\left(x,z\right)= & sgn\left(z\right)\frac{2iW\omega \epsilon }{c^{2}}\frac{\zeta_{\sigma }}{\tilde{E}\left(\omega \pi \right)}\\ & \sum_{n=0}^{N}{\left(-1\right)}^{n}A_{n}\int_{0}^{\infty }du\frac{1-\xi_{u}}{\xi_{u}}\frac{usin(u/2)}{u^{2}-4n^{2}\pi^{2}}\frac{cos(ux/W)}{k_{u}}\times e^{-k_{u}\left|z\right|/W} \end{array}$$

The propagation of surface plasmons is governed by the dispersion relation derived by using the condition of the discontinuity of $B_{y}$ at z=0. For a relatively large $\sigma_{GNDs}$, i.e., $\frac{1-\xi_{u}}{\xi_{u}}=-1$, the discontinuity of $B_{y}$ is reduced to the following dispersion relation:

(13) $$u=-\frac{W\omega (\epsilon_{1}+\epsilon_{2})}{\sigma }$$

where σ is the total conductivity of the GNDs and MNPs arrays at z=0 and $\epsilon_{1}\left(\epsilon_{2}\right)$ is the electric permittivitiy of the dielectric medium in z>0(z<0). Note that the frequency of the surface plasmon polaritons for the system can be determined by solving for the pole of the dispersion relation that is given in terms of geometrical features and optical properties of the system.

To study the properties of the proposed MNPs-GNDs-QDs hybrid-system-based spaser, we focus on the z-component of the electric field, which describes the confined plasmonic field in the direction perpendicular to the interface, since the dipole transitions of the gain medium are only allowed along this direction [31]. Let the wavenumber-dependent amplitude in the field equation be:

(14) $$\sum_{n=0}^{\infty }A_{n}\frac{usin(u/2)}{u^{2}-4n^{2}\pi^{2}}=\epsilon_{u}$$

$\epsilon_{u}$ can also be written in terms of the surface plasmon polaritons energy $\hbar \omega_{u}$ where the field can be quantized by using the Brillouin expression for the field mean energy in the dispersive medium [42]:

(15) $$\frac{1}{4}\left[\int d^{3}r\frac{d(\omega \epsilon (\omega ,r))}{d\omega }{\left|E_{u}\left(r\right)\right|}^{2}+\int d^{3}r\frac{B_{y,u}^{2}\left(r\right)}{\mu^{2}}\right]=\hbar \omega_{u}$$

where μ and ϵ(ω,r) are the magnetic permeability and electric permittivity of the dispersive medium, respectively. The latter can be defined as:

(16) $$\epsilon \left(\omega ,r\right)=\frac{\sigma (\omega )}{\omega }\delta \left(z\right)+\epsilon_{1}\Theta \left(z\right)+\epsilon_{2}\left(1-\Theta \left(z\right)\right)$$

Solving Equation (15) yields the z-component of the quantized plasmonic field of mode $\omega_{u}$ in the z<0 region of the dielectric medium (medium 2) where the gain medium is placed:

(17) $${\hat{E}}_{z,u}=\sqrt{\frac{4\hbar \omega_{u}u}{S_{0}W\left(\epsilon_{1}+\epsilon_{2}\right)\left[-\frac{5}{4}-\frac{2\omega_{u}}{u}\frac{du}{d\omega_{u}}\right]}}sin\left(ux/W\right)e^{k_{2u}z/W}\left({\hat{a}}_{u}^{†}+{\hat{a}}_{u}\right)$$

where ${\hat{a}}_{u}^{†}$ and ${\hat{a}}_{u}$ are the raising and lowering operators of the SPs, respectively. |z| represents the penetration length of plasmonic field that can be adjusted to be the distance between the GNDs layer and the active medium. The area of the GNDs layer is denoted by $S_{0}$, and $du/d\omega_{u}$ included in Equation (17) can be obtained by using the dispersion relation, Equation (13):

(18) $$\begin{array}{ll} \frac{du}{d\omega_{u}}= & -(\epsilon_{1}+\epsilon_{2})W\\ & \left[\frac{2\sigma_{GNDs}+\left[\sigma_{MNPs}-\sigma_{GNDs}\right]\frac{W}{L}-\omega_{u}\left(2E\left(\omega_{u}\right)+F\left(\omega_{u}\right)\frac{W}{L}-2E\left(\omega_{u}\right)\frac{W}{L}\right)}{{\left(2\sigma_{GNDs}+\left[\sigma_{MNPs}-\sigma_{GNDs}\right]\frac{W}{L}\right)}^{2}}\right] \end{array}$$

where for GNDs layer of thickness *t* and Fermi energy $E_{F}$:

(19) $$\begin{array}{ll} E(\omega_{u})= & i\epsilon_{0}t-\frac{i\pi \epsilon_{0}t[\left(A\left(\omega_{u}\right)B{\left(\omega_{u}\right)}^{1/2}-\omega_{u}D\left(\omega_{u}\right)\right]}{A\left(\omega_{u}\right)B\left(\omega_{u}\right)}+\frac{i\epsilon_{0}t\left(1+\pi /2\right)\left(B\left(\omega_{u}\right)-\omega_{u}C\left(\omega_{u}\right)\right)}{B^{2}\left(\omega_{u}\right)}\\ & -\frac{\frac{i\left(1+\pi /2\right)e^{2}E_{F}}{\pi \hbar^{2}\left(\omega_{u}^{2}+\gamma_{G}^{2}\right)}\left[\frac{\left(\hbar^{2}\omega_{u}^{2}+\hbar^{2}\gamma_{G}^{2}\right)-2\hbar^{2}\omega_{u}^{2}}{\left(\hbar^{2}\omega_{u}^{2}+\hbar^{2}\gamma_{G}^{2}\right)}B\left(\omega_{u}\right)-\omega_{u}C\left(\omega_{u}\right)\right]}{B^{2}\left(\omega_{u}\right)}\\ & +\frac{\frac{ie^{2}E_{F}}{\left(\hbar^{2}\omega_{u}^{2}+\hbar^{2}\gamma_{G}^{2}\right)}\left[\frac{\left(\hbar^{2}\omega_{u}^{2}+\hbar^{2}\gamma_{G}^{2}\right)-2\hbar^{2}\omega_{u}^{2}}{\left(\hbar^{2}\omega_{u}^{2}+\hbar^{2}\gamma_{G}^{2}\right)}{\left(A\left(\omega_{u}\right)B\left(\omega_{u}\right)\right)}^{1/2}-\omega_{u}D\left(\omega_{u}\right)\right]}{A\left(\omega_{u}\right)B\left(\omega_{u}\right)} \end{array}$$

(20) $$\begin{array}{ll} F(\omega_{u})= & 2i\epsilon_{0}\epsilon_{d}\left[1+\frac{\left(\frac{-\omega_{p}^{2}}{\omega_{u}^{2}\delta_{M}\epsilon_{d}}-1\right)\left(\frac{\omega_{p}^{2}}{\omega_{u}^{2}\epsilon_{d}}+1\right)+2\frac{\omega_{p}^{2}}{\epsilon_{d}\omega_{u}^{3}}\left(\frac{\omega_{p}^{2}}{\omega_{u}\delta_{M}\epsilon_{d}}-\omega_{u}\right)}{{\left(\frac{\omega_{p}^{2}}{\omega_{u}^{2}\epsilon_{d}}+1\right)}^{2}}\right]tan^{-1}\left(\frac{\gamma_{M}/\omega_{u}}{\frac{\omega_{p}^{2}}{\omega_{u}^{2}\delta_{M}\epsilon_{d}}-1}\right)\\ & +2i\epsilon_{0}\epsilon_{d}\left(\omega_{u}+\frac{\frac{\omega_{u}^{2}}{\omega_{u}\delta M\epsilon_{d}}-\omega_{u}}{\frac{\omega_{p}^{2}}{\omega_{u}^{2}\epsilon_{d}}+1}\right)\times \frac{\frac{-\gamma_{M}}{\omega_{u}^{2}}\left(\frac{\omega_{p}^{2}}{\omega_{u}^{2}\eta_{M}\epsilon_{d}}-1\right)+\frac{2\omega_{p}^{2}\gamma_{M}}{\epsilon_{d}\delta_{M}\omega_{u}^{4}}}{{\left[\frac{\omega_{p}^{2}}{\omega_{u}^{2}\delta_{M}\epsilon_{d}}-1\right]}^{2}\left[1+\frac{{\left(\frac{\gamma_{M}}{\omega_{u}}\right)}^{2}}{{\left(\frac{\omega_{p}^{2}}{\omega_{u}^{2}\delta_{M}\epsilon_{d}}-1\right)}^{2}}\right]} \end{array}$$

(21) $$\begin{array}{ll} C(\omega_{u})= & \frac{\left[2\pi \hbar^{2}\epsilon_{0}\epsilon_{d}t\left(1+\epsilon_{d}\right)\omega_{u}\right]\left[\pi \epsilon_{0}\epsilon_{d}^{2}t\hbar^{2}\left(\omega_{u}^{2}+\gamma_{G}^{2}\right)\right]}{{\left[\pi \epsilon_{0}\epsilon_{d}^{2}t\hbar^{2}\left(\omega_{u}^{2}+\gamma_{G}^{2}\right)\right]}^{2}}\\ & -\frac{\left[-\epsilon_{d}e^{2}E_{F}+\pi \epsilon_{0}\left(1+\epsilon_{d}\right)t\epsilon_{d}\hbar^{2}\left(\omega_{u}^{2}+\gamma_{G}^{2}\right)\right]\left[2\pi \epsilon_{0}\hbar^{2}\epsilon_{d}^{2}\omega_{u}\right]}{{\left[\pi \epsilon_{0}\epsilon_{d}^{2}t\hbar^{2}\left(\omega_{u}^{2}+\gamma_{G}^{2}\right)\right]}^{2}} \end{array}$$

(22) $$D\left(\omega_{u}\right)=\frac{1}{2}{\left[A\left(\omega_{u}\right)B\left(\omega_{u}\right)\right]}^{-1/2}\left[A\left(\omega_{u}\right)\frac{dB}{d\omega_{u}}+B\left(\omega_{u}\right)\frac{dA}{d\omega_{u}}\right]$$

(23) $$A\left(\omega_{u}\right)=\left(\frac{2\pi \epsilon_{0}\omega_{u}\hbar^{2}\left(\omega_{u}^{2}+\gamma_{G}^{2}\right)\left(1-\epsilon_{d}\delta_{G}\right)-e^{2}E_{F}\omega_{u}-2i\epsilon_{0}\hbar^{2}\epsilon_{d}\pi \delta_{G}t\gamma_{G}\left(\omega_{u}^{2}+\gamma_{G}^{2}\right)}{\pi \epsilon_{0}\epsilon_{d}\delta_{G}\hbar^{2}\omega_{u}\left(\omega_{u}^{2}+\gamma_{G}^{2}\right)}\right)$$

(24) $$B\left(\omega_{u}\right)=\frac{-\epsilon_{d}e^{2}E_{F}+\pi \epsilon_{0}\left(1+\epsilon_{d}\right)t\epsilon_{d}\hbar^{2}\left(\omega_{u}^{2}+\gamma_{G}^{2}\right)}{\pi \epsilon_{0}\hbar^{2}\epsilon_{d}^{2}t\left(\omega_{u}^{2}+\gamma_{G}^{2}\right)}$$

**Figure 2:**
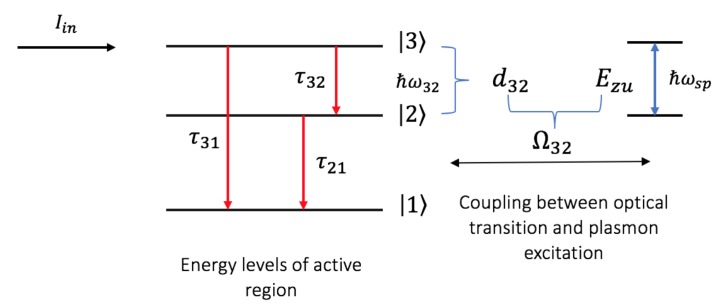
Model of the proposed MNPs-GNDs-QDs hybrid-system-based spaser.

The model of the proposed mid-infrared MNPs-GNDs-QDs hybrid-system-based spaser is shown in Figure 2. Following injection current pumping $I_{in}$, the electrons injected into the upper sub-band (level 3) of the GaN QDs cascade emitter undergo transitions to the lower level (level 2) stimulated by resonant surface plasmons. Subsequently, these electrons relax non-radiatively to the lowest energy level (level 1) to eventually tunnel via the chirped superlattice to the adjacent QD with rate $\gamma_{out}$ [31].

In the presence of a SPs mode resonant with the dipole transition energy $\hbar \omega_{32}$ of the GaN QDs cascade gain medium, energy transfer will occur through the coupling between the dipole transitions of the gain medium and plasmon excitations, leading to the amplification of this mode of SPs. Note, that the number of periods of the QDs cascade emitter that can be included in the energy transfer depends on the penetration length of the plasmonic fields.The dynamics of the system can be investigated by solving the Lindblad master equation through the use of the following Hamiltonian for the u mode:

(25) $$H_{u}=\hbar \omega_{u}{\hat{a}}_{u}^{†}{\hat{a}}_{u}+\frac{1}{2}\hbar \omega_{32}\left(\sigma_{33}-\sigma_{22}\right)-\hbar \left[\Omega_{32}|3\rangle \langle 2|{\hat{a}}_{u}+\Omega_{32}^{*}{\hat{a}}_{u}^{†}|2\rangle \langle 3|\right]$$

where $\Omega_{32}$ is the coupling parameter illustrated in Figure 2 and defined as $d_{32}E_{uz}/\hbar$. $d_{32}$ is the dipole moment of transition |3〉↔|2〉. Since the dipole transition of the GaN QDs cascade is along the z-direction, we consider only the z-component of the plasmonic fields. The two first terms of the Hamiltonian refer to the plasmonic resonator with mode u and the gain medium, respectively. The third term describes the energy transfer between the optical transitions in the gain medium and plasmon excitations. Specifically, $|3\rangle \langle 2|\;{\hat{a}}_{u}$ corresponds to the absorption of a SP in order to make the resonant transition from |2〉 to 〈3|. On the other hand, ${\hat{a}}_{u}^{†}|2\rangle \langle 3|$ represents the emission of a SP to make the transition from |3〉 to 〈2|. The Liouvillian of the system that describes the decay channels is given by [43]:

(26) $$\begin{array}{ll} L_{\rho }= & \frac{\gamma_{31}}{2}\left(\rho \sigma_{33}+\sigma_{33}\rho -2\sigma_{13}\rho \sigma_{31}\right)\\ & +\frac{\gamma_{32}}{2}\left(\rho \sigma_{33}+\sigma_{33}\rho -2\sigma_{23}\rho \sigma_{32}\right)\\ & +\frac{\gamma_{21}}{2}\left(\rho \sigma_{22}+\sigma_{22}\rho -2\sigma_{12}\rho \sigma_{21}\right)\\ & +\gamma_{out}\left(\rho \sigma_{11}+\sigma_{11}\rho \right) \end{array}$$

where $\gamma_{out}$ is the rate of tunneling given in terms of the dimension of QD ($L_{QD}$), and its effective mass ($m_{QD}$) as [37]:

(27) $$\gamma_{out}=\frac{\pi \hbar }{\left(2L_{QD}^{2}m_{QD}\right)exp\left[\frac{2L_{b}\sqrt{2m_{b}H_{b}}}{\hbar }\right]}$$

$L_{b}$ and $m_{b}$ in Equation (21) are the thickness of the barrier and its effective mass, respectively. The height of the potential barrier is denoted by $H_{b}$. With the above consideration, the density matrix equations of the system are given as [44]:

(28a) $${\dot{\rho }}_{032}=-\left[\left(\frac{\gamma_{31}}{2}+\frac{\gamma_{32}}{2}+\frac{\gamma_{21}}{2}\right)+i\left(\omega_{32}-\omega_{sp}\right)\right]\rho_{032}+i\Omega_{32}a_{0u}\left(\rho_{33}-\rho_{22}\right)+\frac{I_{in}}{e},$$

(28b) $${\dot{\rho }}_{031}=-\left(\frac{\gamma_{31}}{2}+\frac{\gamma_{32}}{2}\right)\rho_{031}-i\left[\frac{\omega_{32}}{2}-\left(\omega_{sp}-i\gamma_{21}\right)\right]\rho_{031}+i\Omega_{32}\rho_{021}a_{0u}+\frac{I_{in}}{e},$$

(28c) $${\dot{\rho }}_{021}=i\left[\left(\frac{\omega_{32}}{2}-i\gamma_{21}\right)+i\gamma_{out}\right]\rho_{021}+i\Omega_{32}^{*}a_{0u}^{*}\rho_{031},$$

(28d) $${\dot{\rho }}_{33}=-\left(\gamma_{31}+\gamma_{32}\right)\rho_{33}-\left(i\Omega_{32}\rho_{023}a_{0u}-i\Omega_{32}^{*}\rho_{032}a_{0u}^{*}\right)+\frac{I_{in}}{e},$$

(28e) $${\dot{\rho }}_{22}=\gamma_{32}\rho_{33}-\gamma_{21}\rho_{22}+\left(i\Omega_{32}\rho_{023}a_{0u}-i\Omega_{32}^{*}\rho_{032}a_{0u}^{*}\right),$$

(28f) $${\dot{\rho }}_{11}=\gamma_{31}\rho_{33}+\gamma_{21}\rho_{22}-\gamma_{out}\rho_{11},$$

(28g) $${\dot{a}}_{0u}=\left[i\left(\omega_{32}-\omega_{sp}\right)-\gamma_{sp}\right]a_{0u}-i\rho_{032}\Omega_{32}^{*},$$

where $a_{u}=a_{0u}e^{-i\omega_{sp}t}$, $\rho_{32}=\rho_{032}e^{-i\omega_{sp}t}$, $\rho_{31}=\rho_{031}e^{-i(\omega_{sp}-i\gamma_{21})t}$ and $\rho_{21}=\rho_{021}e^{-\gamma_{21}t}$, with the slowly varying amplitudes $a_{0u}$, $\rho_{032}$, $\rho_{031}$ and $\rho_{021}$. $\gamma_{sp}$ is the damping rate of the SPs. Note that the rate of the injection current, i.e., $I_{in}/e$, where *e* is the charge of an electron, affects the coherence and population terms that are related to the upper level [31]. $\gamma_{ij}$ are the damping rates corresponding to the relaxation times ($\tau_{ij}$) shown in Figure 2.

## 3. Analysis of the Confined Plasmonic Fields near the GNDs Layer

**Figure 3:**
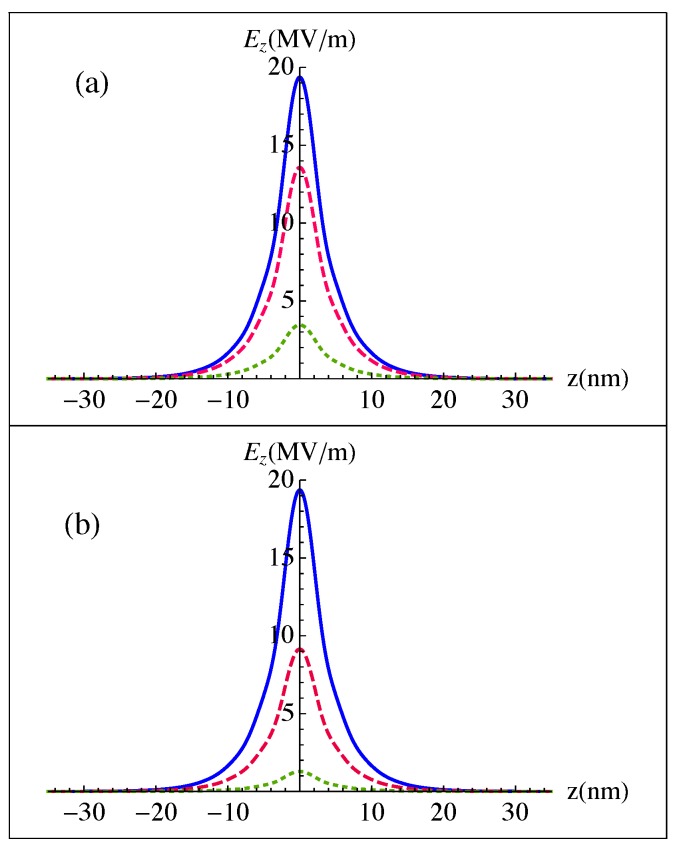
Confined plasmonic fields near the GNDs layer with a thickness of t=0.34 nm, Fermi energy of $E_{F}=1$ eV, mobility of 20,000 cm${}^{2}$/V s and $\delta_{GNDs}=100$ excited by a 10 fs-sech pulse of wavelength $\lambda_{0}=30\;\mu$m, in the presence of the metallic contact, i.e., an Ag NPs lattice of $\delta_{AgNPs}=150$ and (**a**) width of: $W=0.25\lambda_{0}$ (solid), $W=0.5\lambda_{0}$ (dashed) and $W=1.2\lambda_{0}$ (dotted) at x/W=0.45. The two lattices are embedded in a dielectric of ϵ=3. (**b**) width of $W=0.25\lambda_{0}$ for the metallic contact at x/W=0.45. The two lattices are embedded in dielectric medium of ϵ=3 (solid), ϵ=6 (dashed), ϵ=10 (dotted).

This paper aims to study the optical properties of a spaser consisting of a GNDs lattice excited by means of an MNPs one as its resonator and a GaN QDs cascade emitter as its gain medium. Thus, since the QDs emitter has optical transitions only along the direction of the QDs growth, i.e., the *z*-direction, we focus on the z-component of the electric field given by Equation (11), that describes the confined plasmonic fields near the GNDs layer. Figure 3 shows the confined plasmonic field over a few tens of nanometers, corresponding to the penetration length of the plasmonic field, where the sum in Equation (11) is running up to N=20. Clearly, with a 10 fs-sech launching pulse of time dependence given in terms of the time duration of the pulse Δτ as [45],

(29) $$E\left(t\right)={\left(\frac{log(1+\sqrt{2})}{\Delta \tau }\right)}^{1/2}sech\left(\frac{2tlog(1+\sqrt{2})}{\Delta \tau }\right)$$

We get a relatively strong confined plasmonic field of MV/m strength that is significantly enhanced because of the ultrashort pulsed excitation. Moreover, the confined plasmonic fields are enhanced for a relatively small ratio between the width of the metallic contact and the wavelength of the incident radiation, as well as a small dielectric constant of the medium where the two lattices are embedded. This can be attributed to the corresponding limited retardation effects that are responsible for the decay of the confined surface plasmons as an electromagnetic radiation [46].

**Figure 4:**
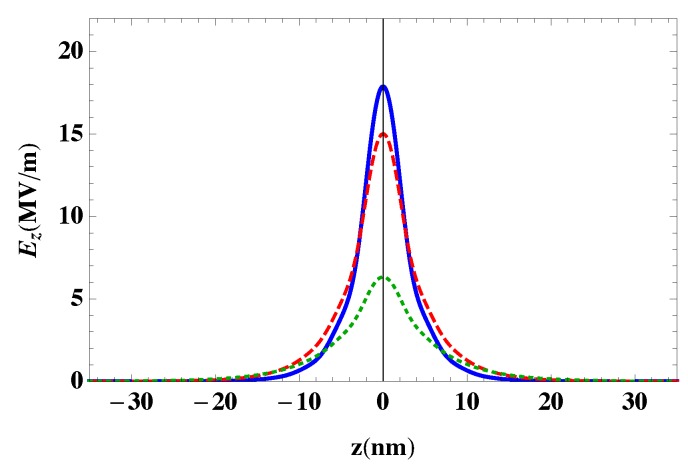
Confined plasmonic fields near the GNDs layer with thickness of t=0.34 nm, Fermi energy of $E_{F}=1$ eV and mobility of 20,000 cm${}^{2}$/V s, excited by a 10 fs-sech pulse of $\lambda_{0}=30\;\mu$m, in the presence of metallic contact, i.e., an Ag NPs lattice of width $W=0.25\lambda_{0}$ at x/W=0.45 with $\delta_{GNDs}=50-\delta_{AgNPs}=100$ (solid), $\delta_{GNDs}=100-\lambda_{AgNPs}=100$ (dashed) and $\delta_{GNDs}=200-\delta_{AgNPs}=100$ (dotted). The two lattices are embedded in a dielectric of ϵ=3.

Additionally, we examine the effect of the packing density of the GNDs and Ag NPs lattices on the confinement of the plasmonic fields near the GNDs layer, as illustrated in Figure 4. We observe that a relatively strong confined plasmonic field is obtained when the packing density of the Ag NPs lattice is larger than that of the GNDs one. The confined plasmonic field decreases as the difference of packing density between the two lattices decreases. We observe a significant decrease of the confined plasmonic field for the case of $\delta_{GNDs}>\delta_{AgNPs}$. This is reasonable, since the plasmonic fields are enhanced for a large $\delta_{AgNPs}/\delta_{GNDs}$ corresponding to a highly conductive stripe. Note, that the conductivities of the two lattices are increased for a large packing density [39].

**Figure 5:**
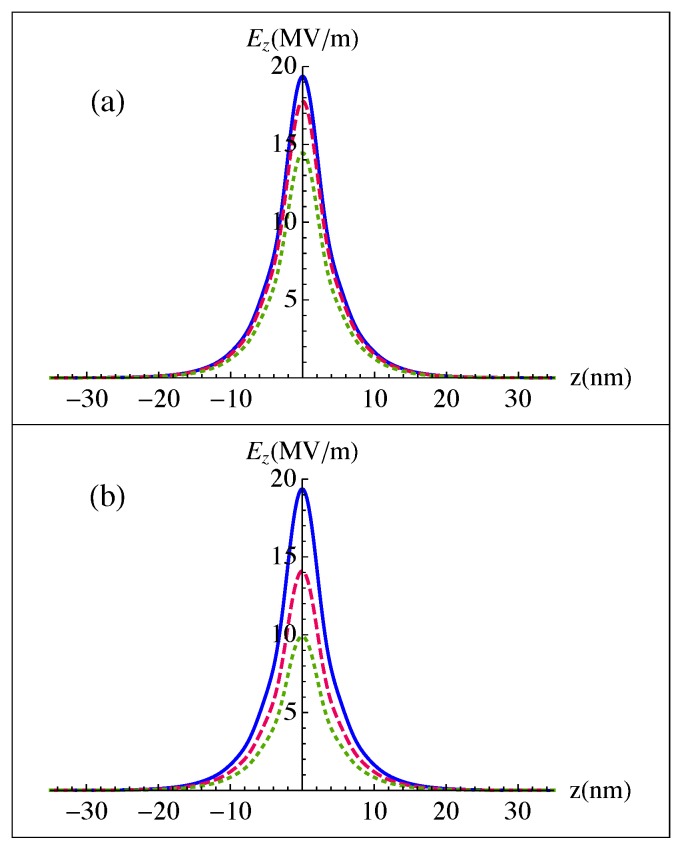
Confined plasmonic fields near the GNDs layer with a mobility of 20,000 cm${}^{2}$/Vs excited by a 10 fs-sech pulse of $\lambda_{0}=30\mu$m, in the presence of metallic contact, i.e., an Ag NPs lattice of width $W=0.25\lambda_{0}$ at x/W=0.45 with $\delta_{GNDs}=100-\delta_{AgNPs}=150$. The two lattices are embedded in a dielectric of ϵ=3. The thickness and doping level of GNDs ar: (**a**) t = 0.34 nm, $E_{F}=1$ eV (solid), $E_{F}=0.7$ eV (dashed) and $E_{F}=0.4$ eV (dotted). (**b**) $E_{F}=1$ eV, t=0.34 nm (solid), t=0.55 nm (dashed), t=0.6 nm (dotted).

Figure 5 shows the effect of the thickness of the GNDs (t) and their doping levels, which are defined by the Fermi energy $\left(E_{F}\right)$, on the confined plasmonic fields. Clearly, the latter are enhanced for a relatively small thickness of GNDs with large doping levels that lead to a relatively large bulk plasma frequency for graphene, $\omega_{p,G}^{2}=\left(e^{2}E_{F}\right)/\left(\pi \hbar^{2}\epsilon_{0}\epsilon t\right)$. It can be seen that the penetration length is not affected by the thickness of the GNDs and their doping levels. Additionally, the confined plasmonic field near the GNDs layer is sensitive to the thickness of the GNDs more than to the doping level.

## 4. Analysis of the Dynamics of the Proposed MNPs-GNDs-QDs Hybrid System-Based Spaser

Unlike the conventional lasers and amplifiers in quantum electronics, spasers have an inherent feedback provided by plasmonic structures that typically cannot be removed [2]. Thus, a spaser will develop the accumulation of a large number of coherent SPs, leading to a continuous wave (CW) regime where the gain compensates exactly for the losses with zero net amplification. One way to operate a spaser as a plasmonic amplifier is to consider the transient regime, recalling the fact that the establishment of the CW regime requires a relatively long time [20]. Thus, we consider the transient amplification of SPs during hundreds of femtoseconds, a time interval smaller than the relaxation times of the gain medium and SPs. The transient dynamics of the spaser can be directly obtained by the numerical solution of the density matrix equations (Equation (22)).

At first, we solve numerically for the pole of the dispersion relation of SPs for the planar structure given by Equation (13). The parameters of the system are adjusted to get the SPs mode energy to be resonant with that of the GaN QDs cascade emitter. Specifically, for a GNDs monolayer of a thickness of 0.34 nm, Fermi energy of 0.7 eV and mobility of $10^{4}$ cm${}^{2}$/V s with the GNDs lattice (Ag NPs lattice) embedded in a dielectric medium of dielectric constant 3 (12) with packing density 100 (150), we get an SPs mode energy of 41.5 meV, which is resonant with the laser transition of GaN QD terahertz cascade laser [37]. The GaN QD is modeled as a three-level system of cascade configuration, where the energy spacing between the two lower levels is resonant with longitudinal optical phonon scattering, leading to the fast depopulation of the lower state of the laser transition, which makes this quantum emitter attractive as a gain medium. The relaxation rates of the gain medium are set to be $\gamma_{31}=7.14\times 10^{10}s^{-1}$, $\gamma_{32}=7.7\times 10^{10}s^{-1}$ and $\gamma_{21}=2.2\times 10^{12}s^{-1}$ [37], whereas that of the SPs corresponding to $E_{F}=0.7$ eV is $\gamma_{sp}=7.12\times 10^{11}s^{-1}$. The rate of injection is set to be $10^{10}s^{-1}$ and the rate of tunneling is calculated with a GaN QD of dimension $L_{QD}=4$ nm and an effective mass of $0.2m_{0}$ with an AlGaN barrier of $L_{b}=3$ nm and $m_{b}=0.3m_{0}$, correspondingly [47]. The dipole moment of the gain medium is taken as 2.5e nm [47] and the area of the GNDs layer is set to be 75 μm${}^{2}$.

**Figure 6:**
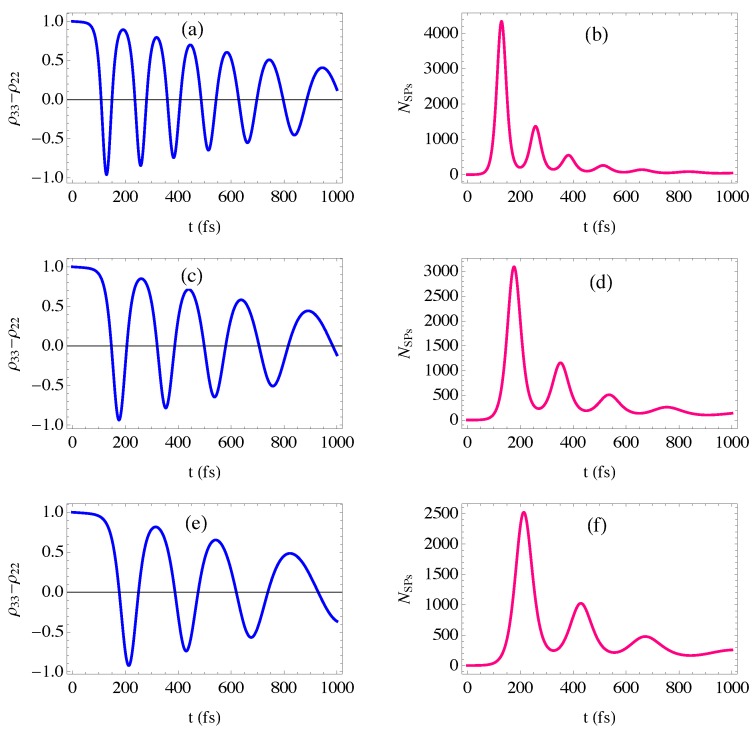
Ultrafast dynamics of the proposed MNPs-GNDs-QDs hybrid-system-based spaser: (**a**,**c**,**e**) the temporal behavior of the population inversion; (**b**,**d**,**f**) the time evolution of SPs population in the spasing mode of energy 41.5 meV with metallic width (a,b): $W=0.2\lambda_{0}$, (c,d): $W=0.4\lambda_{0}$ and (e,f): $W=0.6\lambda_{0}$. $\lambda_{0}=30$μm is the wavelength of a 1ps-pulse excitation.

The plasmonic components are launched by a 1ps-sech pulse that is shorter than the relaxation times of the gain medium, so that the slowly varying approximation is valid [48] and our calculations for the energy transfer between the plasmonic excitations and the optical transitions in the gain medium are simplified. The dynamics of energy transfer between the SPs and the optical transitions of the gain medium are shown in Figure 6 by numerically solving for the time-dependent density matrix equations (Equation (22)) with initial conditions $\rho_{33}\left(0\right)=a_{0u}^{*}\left(0\right)=1$; that is, electrons are initially in the upper level (level 3) and a resonant SP mode is present to induce the transition to the lower level (level 2). The penetration length of the plasmonic field is set to be equivalent to one period of the GaN QDs cascade emitter, i.e., z=17 nm [37].

It can be seen that transient amplification of the SPs mode of energy 41.5 meV is obtained over hundreds of femtoseconds before they are damped depending on the relaxation rates of the SPs and the gain medium. It is remarkable that at the instant the number of SPs is maximized, the population inversion reaches its minimum, implying that the energy of optical transition is completely transferred to the SPs to be amplified. Interestingly, the amplification of SPs is significantly affected by the width of the metallic contact, emphasizing the important role of the metal in the energy transfer as shown in [32]. Specifically, for a large width of the metallic contact, the amplification of SPs is decreased and the time corresponding to the maximum number of SPs is displaced to later values. This can be attributed to the retardation effects that dominate as the width of the metallic contact approaches the wavelength of the incident radiation, leading to the decay of the SPs [49].

**Figure 7:**
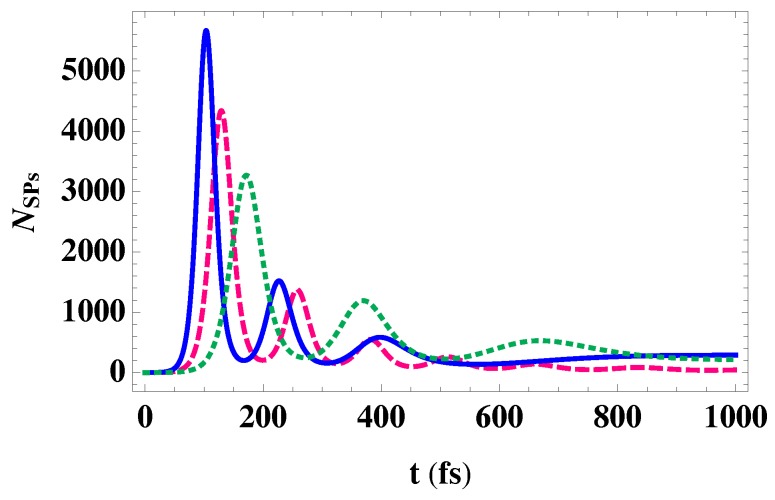
Ultrafast dynamics of the SPs in the proposed MNPs-GNDs-QDs hybrid-system-based spaser with a metallic contact of width $W=0.3\lambda_{0}$, induced by a sech-pulse excitation of wavelength $\lambda_{0}=30\;\mu$m and time duration of Δτ=400fs (solid), Δτ=600fs (dashed) and Δτ=2ps (dotted).

Figure 7 shows the effect of the time duration of the normalized sech-pulse used for launching SPs on the dynamics of the SPs mode of energy 41.5 meV. It can be seen that the amplification of SPs is enhanced for a small time duration of the launching pulse that is associated with a relatively large intensity. This enhancement is due to the large local field enhancement induced near the surface of plasmonic materials with pulsed excitation [50]. Interestingly, the transient amplification occurs over the hundreds of femtoseconds, a time duration smaller than the relaxation time of the upper level, so that the spontaneous emission noise can be avoided. Moreover, the spasing threshold of our proposed spaser consisting of a GNDs-MNPs plasmonic system and a GaN QDs cascade emitter that are separated by a distance *l* can be easily estimated by a comparison between losses and gains: $\hbar^{2}{\left|\Omega_{32}\right|}^{2}e^{-2ql}\geq \Gamma_{SP}\Gamma_{32}$. Interestingly, the spasing threshold of the proposed spaser is smaller than that demonstrated for the graphene spaser proposed by Apalkov et al. [12], due to the relatively small inter-sub-band-polarization relaxation width of the gain medium $(\Gamma_{32}=50\;\mu$eV ) and the relatively low damping width of the SPs $(\Gamma_{SP}=470\;\mu$eV). Specifically, the spasing condition is given in terms of the time relaxation of plasmons by [12]:

(30) $$\tau \geq \tau_{min}=\frac{4e^{2}\Gamma_{32}E_{F}e^{2ql}}{\hbar^{2}\omega_{u}^{3}k_{0}^{2}{\left|d_{32}\right|}^{2}}$$

where *q* is the wave number of plasmons and $\Gamma_{32}$ is the relaxation width of the QD. The distance between the graphene and the QD is denoted by *l*. $k_{0}$ is the effective Fermi wave vector that can be easily calculated by knowing the energy of plasmons $\left(\hbar \omega_{q}\right)$, which is chosen to be at resonance with the QD transition frequency in order to facilitate the energy transfer between plasmons in graphene and excitons in QD, which is essential for spasing. Therefore, the operating frequency of our proposed spaser is limited by the transition frequency of the QD. In fact, our focus is on enhancing the amplification and not on introducing broad tunability. For the following parameters: $\Gamma_{32}=50\;\mu$eV, $E_{F}=1$ eV, l=17 nm, $k_{0}=1.1$ nm${}^{-1}$, $\hbar \omega_{q}=41.5$ meV, $d_{32}=2.5$ enm, we get an extremely short minimum relaxation time of graphene plasmons, i.e., $\tau_{min}=435$, which corresponds to a quality of factor $Q_{min}=\left(\omega_{q}\tau_{min}\right)/2=0.013$. The obtained quality factor is lower than the one obtained by Apalkov et al. [12], implying that the spasing in our proposed hybrid nano-system is relatively easy to achieve.

Clearly, the transient regime represents the region where the spaser can act as a plasmonic amplifier with a gain exceeding the losses, achieving an amplification of a selected mode of SPs that can be controlled by the width of metallic contact and the time duration of the launching pulse, leading to an enhancement of plasmonics.

## 5. Conclusions

In this paper, we have studied the properties of the proposed MNPs-GNDs-QDs hybrid-system-based spaser. Firstly, we investigated the confined plasmonic fields near the interface of the GNDs lattice with a metallic contact, represented by an Ag NPs lattice, induced by the launching pulse that has its linear polarization along the interface between the GNDs layer and the dielectric environment. We have found that the plasmonic field can be enhanced for a small width of the metallic contact and a small dielectric constant of the surrounding medium, because then the retardation effects are limited. Moreover, the confined plasmonic field is enhanced for a small thickness of the GNDs and large doping levels that lead to a relatively large density of charge carriers. A large packing density in the MNPs lattice compared to that of the GNDs can also significantly enhance the confined plasmonic fields.

Subsequently, we have studied the dynamics of the proposed spaser with a GaN QDs cascade emitter chosen to be the gain medium. A novel MNPs-GNDs-QDs hybrid-system-based spaser is obtained with a transient amplification of the SPs mode, providing a plasmonic amplifier. The number of SPs that determines the intensity of the obtained plasmonic field is significantly affected by the width of the metallic contact, emphasizing the important role of the MNPs lattice in the spasing process. In particular, the presence of an MNPs lattice provides more options for controlling and enhancing the amplification of the plasmonic fields.

Interestingly, the various factors in our scheme, which was identified to affect the amplification of the plasmonic fields, act cooperatively and cumulatively. They are within reach of present-day materials technology, and various combinations of them, within the theoretically identified limits, could result in plasmonic amplifiers with a low spasing threshold and significant amplification. This feature adds a degree of flexibility in the realization of the herein proposed plasmonic amplifier. Therefore, the proposed controllable SPs amplification scheme could prove useful for various interesting applications, such as stand-alone components or gain sections integrated with other plasmonic elements to compensate for losses and improve the performance of plasmonic devices.
